# Intravenous BCG vaccination in non-human primates induces superior serum antibody titers with enhanced avidity and opsonizing capacity compared to the intradermal route

**DOI:** 10.1016/j.vaccine.2024.126444

**Published:** 2024-12-02

**Authors:** Marco Polo Peralta Alvarez, Keya Downward, Andrew White, Stephanie A. Harris, Iman Satti, Shuailin Li, Alexandra Morrison, Laura Sibley, Charlotte Sarfas, Mike Dennis, Hugo Redondo Azema, Sally Sharpe, Helen McShane, Rachel Tanner

**Affiliations:** a Jenner Institute, Nuffield Department of Medicine, University of Oxford, Oxford OX3 7DQ, UK; b Laboratorio Nacional de Vacunologia y Virus Tropicales, Departamento de Microbiologia, Escuela Nacional de Ciencias Biologicas, Instituto Politecnico Nacional, Ciudad de Mexico 11350, Mexico; c UK Health Security Agency, Porton Down, Salisbury SP4 0JG, UK; d Department of Biology, University of Oxford, Oxford OX1 3RB, UK

**Keywords:** IV BCG, Antibody, Opsonization, Avidity, Tuberculosis, Vaccine

## Abstract

A new and more effective tuberculosis (TB) vaccine is urgently needed, but development is hampered by the lack of validated immune correlates of protection. Bacillus Calmette Guérin (BCG) vaccination by the aerosol (AE) and intravenous (IV) routes has been shown to confer superior levels of protection from challenge with *Mycobacterium tuberculosis* (*M.tb*) in non-human primates (NHP) compared with standard intradermal (ID) administration. This finding offers a valuable opportunity to investigate which aspects of immunity are associated with improved control of *M.tb* and may represent biomarkers or correlates of protection. As TB vaccine research to date has focused largely on cellular immunity, we aimed to better characterize the poorly-understood serum antibody response to BCG administered by different routes of vaccination in NHP. We demonstrate superior *M.tb*-specific IgG, IgA, and IgM titers in serum following IV BCG vaccination compared to the ID or AE routes. We also observe improved capacity of IgG induced by IV BCG to opsonize the surface of mycobacteria, and report for the first time that *M.tb*-specific IgG from IV BCG vaccinated animals is of higher avidity compared with IgG from ID or AE BCG vaccinated animals. Notably, we identified a significant correlation between IgG avidity and measures of protection from aerosol *M.tb* challenge. Our findings highlight a potential role for antibodies as markers and/or mediators of the superior vaccine-induced protection IV BCG confers against TB and suggest that quality, as well as quantity, of antibodies should be considered when developing and evaluating TB vaccine candidates.

## Highlights

• Superior serum antibody titers following IV *vs.* ID or aerosol BCG vaccination.
• IgG induced by IV BCG has improved mycobacterial opsonization *vs.* ID or aerosol BCG.
• Higher avidity *M.tb*-specific IgG following IV *vs.* ID or aerosol BCG vaccination.
• Association between IgG avidity and protection from aerosol *M.tb* challenge.

## 1 Introduction

Tuberculosis (TB), caused by *Mycobacterium tuberculosis* (*M.tb*), is the leading cause of mortality due to a single pathogen and remains a major global health problem [1,2]. Bacillus Calmette Guérin (BCG) is a live-attenuated vaccine that is the result of cumulative mutations by serial passage of *Mycobacterium bovis*, and is the only licensed TB vaccine. BCG is widely administered intradermally (ID) close to the time of birth and provides an effective prophylaxis against severe forms of TB in infants and young children [3]. However, efficacy against pulmonary TB, the most common form of disease, is notoriously variable (nil to 80 %) depending on geographical region. The poorest efficacy is seen in many TB endemic countries most in need of protection [[4], [5], [6]]. A new, more effective TB vaccine that improves upon, boosts, or replaces BCG is urgently needed. However, development is hampered by a lack of validated immune correlates of vaccine-mediated protection from TB.

Since the 1970s it has been suggested that administration of BCG by the aerosol (AE) or intravenous (IV) routes of administration could enhance protection against TB in non-human primates (NHPs) [[7], [8], [9], [10]]. Antigens need to reach secondary lymphoid organs such as lymph nodes to effectively initiate adaptive immune responses. The route of exposure likely influences the kinetics and efficiency of antigen trafficking to these organs, thus affecting antigen-specific immunogenicity [11]. Mucosal tissue contains unique immune cell populations that can generate distinct responses [12], and intranasal (IN) BCG vaccination was found to provide better protection against *M.tb* challenge than ID BCG vaccination in mice, particularly in the lungs [13]. Aerosol (AE) vaccination is another promising mucosal alternative which more closely mimics the route of natural infection. In preclinical studies, AE BCG vaccination has been found to be safe and immunogenic [14]. Immunization with either AE BCG or mucosal attenuated *M.tb* is associated with improved immunogenicity and efficacy compared with ID BCG in NHPs [15,16]. Moreover, Dijkman et al. have demonstrated that endobronchial instillation of BCG successfully prevents *M.tb* infection and TB disease in NHPs following ultra-low dose exposure to *M.tb* [17]. BCG delivered by the AE route has recently been shown to be well-tolerated and induced potent Th1 immunity in the lung and systemic circulation in a Phase I clinical trial [18].

There has been a recent resurgence of interest in IV BCG. In 2016, Sharpe et al. compared the efficacy of BCG administered ID *vs.* ID with an IT boost and *vs.* the IV route in NHPs, showing that IV BCG conferred improved protection following aerosol *M.tb* challenge [19]. This was validated by an independent comprehensive study by Darrah et al. in 2020, demonstrating unprecedented levels of protection following IV BCG vaccination with 6 out of 10 animals showing no detectable *M.tb* infection [20]. Although IV BCG may not be an easily deployable strategy in human infants, it represents a valuable model for identifying immune correlates of protection from TB to direct rational vaccine development.

Study of the immune mechanisms underlying the superior protection conferred by AE or IV BCG has focused largely on the cellular response, and the role of antibodies in protection from TB has been under-studied. However, individuals with latent TB infection (LTBI) who are considered to have some degree of protection, have antibodies with distinct glycosylation patterns and enhanced functional responses compared with those from active TB patients [21]. Furthermore, antibodies isolated from *M.tb*-exposed but uninfected healthcare workers can confer protection against *M.tb* challenge when transferred to mice [22].

Evidence for antibody-mediated protection following BCG vaccination remains equivocal [23], but in a *post-hoc* analysis, levels of Ag85A-specific IgG were associated with reduced risk of TB disease in BCG-vaccinated South African infants [24]. Superior antibody responses have been reported following AE and IV BCG vaccination compared with the ID route [14,20], and robust IgM responses following IV BCG have been associated with prevention of *M.tb* infection in NHPs [25]. We set out to further characterize the antibody response to AE and IV BCG in independent NHP cohorts, with a focus on IgG opsonizing capacity and avidity which have not previously been considered in this context.

## 2 Methods

### 2.1 Non-human primate studies

#### 2.1.1 Experimental animals

Rhesus macaques of Indian genotype aged 3–12 years were obtained from an established closed UK breeding colony. Absence of *M.tb* infection and IFN-γ mediated cell-mediated immunity to mycobacterial antigens, was confirmed by *ex-vivo* IFN-γ ELISpot (MabTech, Nacka. Sweden) measuring responses to PPD (SSI, Copenhagen, Denmark) and pooled 15-mer peptides of ESAT6 and CFP10 (Peptide Protein Research LTD, Fareham, U.K.). The animals were housed in compatible social groups and cared for in accordance with the UK Animals (Scientific Procedures) Act 1986, the Home Office (UK) Code of Practice for the Housing and Care of Animals Bred, Supplied or Used in Scientific Purposes (2014), and the National Committee for Refinement, Reduction and Replacement (NC3Rs) Guidelines on Primate Accommodation, Care and Use, August 2006. Animals were sedated by intramuscular injection of ketamine hydrochloride (Ketaset, 100 mg/ml, Fort Dodge Animal Health Ltd., Southampton, UK; 10 mg/kg) for procedures requiring removal from their housing. None of the animals had been used previously for experimental procedures and each socially compatible group was randomly assigned to a particular study treatment. Animal procedures and study designs were approved by the UK Health Security Agency (formally Public Health England), Porton Down Ethical Review Committee, authorized under an appropriate UK Home Office project license, and performed under authorisation through the appropriate national laws and regulations, in compliance with European Directive 201/63/EU and its implementations in national legislation. Stored serum samples were used from six historical NHP studies to avoid the use of additional animals (Table 1). Analysis stratified by sex was not possible due to small numbers of animals (limited for ethical reasons) which were predominantly male.

**Table 1:** Summary of studies from which NHP samples were obtained.

Study code	Species, age and sex	Groups from which samples were used	BCG source	Reference
Study 1	Rhesus macaque, 3–4.5 years, males	Naïve unvaccinated (*n* = 6) ID BCG 2–8 × 10^5^ CFU (*n* = 6) ID BCG 2–8 × 10^5^ CFU + IT BCG 2–8 × 10^6^ CFU (n = 6) IV BCG 2–8 × 10^6^ CFU (n = 6)	Strain 1331 (SSI, Copenhagen, Denmark)	Sharpe et al. [19]
Study 2	Rhesus macaque, 3.8 years, males	ID BCG 2–8 × 10^5^ CFU (n = 6) AE BCG 1 × 10^7^ CFU BCG intended dose (n = 6)	Strain 1331 (SSI, Copenhagen, Denmark)	White et al. [16]
Study 3	Rhesus macaque, 4.5 years, males	ID BCG 2–8 × 10^5^ CFU (*n* = 8)	Strain 1331 (1st WHO reference reagent, NIBSC, UK)	White et al. [47]
Study 4	Rhesus macaque, 10–12 years, 1 male and 5 females	IV BCG 2–8 × 10^6^ CFU (n = 6)	Strain 1331 (AJ Vaccines, Copenhagen, Denmark)	Morrison et al. [48]
Study 5	Rhesus macaque, 6 years, 9 males and 3 females	AE BCG 1 × 10^7^ CFU intended dose (*n* = 12)	Strain 1331 (AJ Vaccines, Copenhagen, Denmark)	n/a
Study 6	Rhesus macaque	Naïve unvaccinated (n = 6)	n/a	n/a

#### 2.1.2 BCG vaccination

BCG vaccinations were performed using BCG Danish strain 1331 (for origin see Table 1), prepared according to the manufacturer's instructions by addition of 1 ml Sauton's diluent to a lyophilised vial. Serum was available from animals that were either unvaccinated controls (*n* = 12, Studies 1 and 6) or received 2–8 × 10^5^ CFU of BCG ID into the upper arm under sedation (*n* = 20, Studies 1–3). Serum was also available from animals that received BCG administered into the lung using an Omron MicroAir mesh nebuliser (Omron Healthcare United Kingdom Ltd., Milton Keynes, United Kingdom) (*n* = 18, Studies 2 and 5). In Studies 2 and 5, 2–8 × 10^6^ CFU and 1 × 10^7^ CFU respectively was loaded into the nebuliser, with the delivered dose estimated to be equivalent to a standard adult ID dose (2–8 × 10^5^ CFU) after taking into account the expected losses in viable BCG titer (∼1 log CFU) associated with the aerosol delivery process. For the IV BCG vaccinated animals, 2–8 × 10^6^ CFU of BCG was delivered IV into the femoral vein of the left leg (*n* = 12, Studies 1 and 4). One group of animals received 2–8 × 10^5^ CFU BCG ID followed by a second vaccination with 2–8 × 10^6^ CFU BCG intratracheally (IT) 12 weeks later (*n* = 6, Study 1). For the IT boost, 1 ml of BCG was delivered using an endotracheal catheter gauge 8 FG inserted into the lung to a depth of 15 cm, with the catheter flushed through using 0.5 ml sterile PBS [19]. For inter-group comparisons, the ID and ID + IT groups are combined at week 8 because at this point they had both received the same treatment (ID BCG only). The ID + IT group received an IT boost at week 12 so are plotted separately at week 20. Further details of the studies from which these samples were derived are provided in Table 1. Not all samples were available for all assays due to limited volumes. Due to small numbers, serum samples from different studies were combined in a single group where the species, route and dose of BCG and sampling time-points were consistent.

#### 2.1.3 *M.tb* challenge

In Study 1, animals were challenged by exposure to a target dose of 100 CFU of aerosolised *M.tb* Erdman strain K01 (BEI resources) delivered into the lungs at 21 weeks after primary vaccination. Mono-dispersed bacteria in particles of mean diameter 2 μm were generated using a 3-jet Collision nebuliser (BGI Incorporated, MA, USA) and delivered to the nares of sedated animals placed in a ‘head-out’ plethysmography chamber (Buxco, Wilmington, NC, USA) *via* a veterinary anaesthesia mask (modified to permit the flow of aerosol over the nose) in conjunction with a modified Henderson apparatus. This allowed aerosol to be delivered simultaneously with the measurement of respiration rate and volume. One animal from each group was exposed in sequence to minimise potential confounders in order of treatment. Animals were monitored daily for behavioural abnormalities including depression, aggression, withdrawal, changes in feeding pattern, altered respiration rate and coughing. Animals were weighed, had rectal temperature measured and were examined for gross abnormalities whenever procedures were conducted. Humane endpoints were determined by experienced primatology staff and based on a predetermined combination of adverse indicators: depression or withdrawn behaviour, abnormal respiration (dyspnoea), loss of 20 % of peak post-challenge weight, ESR levels elevated above normal (>20 mm), haemoglobin level below normal limits (<100 g/dl), increased temperature (>41 °C) and severely altered thoracic radiograph. Outcome measures were time of disease control, lung pathology, total pathology, lung lesion count, lesion:lung ratio, % weight loss, ESR and X-ray score, the results of which have been reported elsewhere [19].

### 2.2 Standardized indirect enzyme-linked immunosorbent assays (ELISAs)

96-well NUNC MaxiSorp plates (Thermo Fisher) were coated with 50 μL of antigen solution diluted in Dulbecco's Phosphate Buffered Saline (DPBS) and incubated for 16-18 h at 4 °C. The antigen mixtures used for coating were *M.tb* whole cell lysate (WCL) (1 μg/ml) and *M.tb* purified protein derivative (PPD) (5 μg/ml). Plates were then blocked with 100 μl of casein-PBS blocker solution for 1 h and incubated for 2 h with 50 μl of NHP serum diluted 1:100 in casein. Plates were then incubated for 1 h with 50 μl/well of a 1:1000 dilution in casein of either goat anti-monkey IgG γ-chain-specific, goat anti-monkey IgM μ-chain-specific or goat anti-monkey IgA α-chain-specific secondary antibodies conjugated to alkaline phosphatase (AP). Finally, 100 μl of 4-Nitrophenyl phosphate disodium salt hexahydrate alkaline phosphatase substrate (pNPP) developing solution was added, and plates were subsequently read every 30 min. The optical density reading of the plates at 405 nm was performed using an Elx808 microplate reader (BioTek Instruments). Plates were washed 4× in PBS-Tween and tapped dry between each step, except after blocking where the washing was not performed.

The standard curve used on each plate was derived from a pool of NHP sera previously established to contain a high-titer of *M.tb*-specific antibodies. A 1:100 dilution of the standard pool was used in a two-fold serial dilution to produce ten standard points that were assigned arbitrary ELISA units. The optical density values of the standard points were fitted to a four-parameter hyperbolic curve against the arbitrary ELISA units using Gen5 software (version 3.04; BioTek Instruments, Winooski, VT, USA), and the parameters from the standard curve were used to convert absorbance values of individual test samples into ELISA units. Each ELISA plate consisted of the samples and internal positive control (1:800 dilution of the standard pool, corresponding to standard 4) in triplicate, ten standard points in duplicate, and four blank negative control wells.

### 2.3 Opsonization assay

#### 2.3.1 IgG purification

IgG fractions from 300 μl serum were purified by affinity chromatography on HiTrap Protein G High Performance columns according to manufacturer's instructions (GE Healthcare, United States). The IgG fraction was eluted with 430 μl of 200 mM glycin buffer (pH 2.0; 0.2 ml/min flow) and neutralized at pH 7.0 with 1 M Tris-HCl buffer pH 11.0.

#### 2.3.2 Preparation of BCG-GFP

BCG Montreal (ATCC 35735) containing pEGFP cloned under the control of mycobacterial 19 kDa promoter was kindly provided by Professor Rajko Reljic from St George's University of London, whereinafter designated as BCG-GFP. Cryopreserved BCG-GFP was thawed and grown in medium containing Middlebrook's 7H9 broth (BD Biosciences, UK) supplemented with 10 % OADC enrichment (BD Biosciences, UK), 0.2 % glycerol and 0.05 % tyloxapol at 37 °C, in aerobic conditions, on a shaker at 200 rpm until it reached log phase and an OD of 1.0 by spectrophotometry, which is equivalent to ∼1 × 10^7^ CFU.

#### 2.3.3 Measurement of opsonizing capacity

After harvesting BCG-GFP by centrifugation at 3000*g* for 15 min and washing twice with PBS, bacterial clumps were dissociated by sonication for 10 min and dispersion through a 27g needle. A single cell suspension of 5 × 10^6^ CFU BCG-GFP was incubated for 2 h at room temperature in 2 ml of sterile PBS with 25 μg, 50 μg, or 100 μg, of purified total IgG. BCG was washed twice by centrifuging at 4000*g* for 10 min and re-suspending in sterile PBS, labelled with anti-monkey IgG Fc antibody (V450 fluorochrome, BD Biosciences, UK) for 30 min, and then fixed with 4 % paraformaldehyde for 20 min. Percentage opsonization was defined as the percentage of IgG+ BCG-GFP by flow cytometry gated by GFP+ bacilli.

### 2.4 Avidity assay

96-well NUNC MaxiSorp plates (high protein binding) (Thermo Fisher, United States) were coated with 50 μL of antigen solution diluted in DPBS and incubated for 16-18 h at 4 °C. PPD was used for coating at 5 μg/ml. Plates were then blocked with 100 μl of casein-PBS blocker solution for 1 h and incubated for 2 h with 50 μL of 1:100 NHP serum diluted in casein. 50 μl of the chaotropic agent sodium thiocyanate (NaSCN), was added in increasing concentrations to the samples and incubated for 15 min at room temperature. In all cases NaSCN concentrations ranged from 0 M to 4 M. Plates were then incubated for 1 h with 50 μl/well of 1:1000 dilution of goat anti-monkey IgG γ-chain-specific secondary antibody conjugated to AP. Finally, 100 μl of pNPP developing solution was added. Plates reached an initial OD of 1.0 after approximately 30 min. The reading was performed at a wavelength of 405 nm using an Elx808 microplate reader (BioTek Instruments). Plates were washed 4× in PBS-Tween and tapped dry between each step, except after blocking where the washing was not performed. For each sample data the response was normalised from 0 to 100 % using GraphPad Prism software v.10. Then, the IC_50_ for each sample was calculated.

### 2.5 Mycobacterial growth inhibition assay (MGIA)

The NHP direct MGIA was performed as previously described [26]. 3 × 10^6^ PBMC and ∼ 500 CFU BCG Pasteur in a total volume of 480 μl RPMI (containing 2 mM l-glutamine and 25 mM HEPES), plus 120 μl autologous serum matched to animal and time-point were added per well of a 48-well plate (total volume 600 μl/well). Co-cultures were incubated at 37 °C for 96 h and then transferred to 2 ml screw-cap tubes and centrifuged at 15,300*g* for 10 min. During this time, 500 μl sterile water was added to each well to lyse adherent monocytes and release intracellular mycobacteria. Supernatants were carefully removed from the 2 ml screw-cap tubes by pipetting, and water from the corresponding well added to the remaining pellet. Tubes were pulse vortexed and the lysate transferred to BACTEC MGIT tubes supplemented with PANTA antibiotics and OADC (Becton Dickinson, UK). At the end of the protocol, MGIT tubes were placed on the BACTEC 960 machine (Becton Dickinson, UK) and incubated at 37 °C until the detection of positivity by fluorescence. On day 0, duplicate direct-to-MGIT inoculum control tubes were set up by inoculating supplemented BACTEC MGIT tubes with the same amount of mycobacteria as the samples. The time to positivity (TTP) read-out was converted to log_10_ CFU using stock standard curves of TTP against inoculum volume and CFU. Results are presented as ‘normalised mycobacterial growth’ which is equal to (log_10_ CFU of sample – log_10_ CFU of inoculum control) to correct for variation in inoculum between mycobacterial stocks, batches and aliquots. ‘Vaccine response’ (VR) following BCG vaccination was calculated as (post-vaccination normalised growth – baseline normalised growth) and is presented as Δlog_10_ CFU.

### 2.6 Statistical analysis

Data were analyzed using GraphPad Prism v.10 and SPSS v.29.0. Due to small sample sizes, non-parametric statistical tests were used throughout. Longitudinal data was analyzed using a Friedman test with Dunn's correction for multiple comparisons (all time-points *vs.* baseline). When comparing two conditions, a Wilcoxon signed-rank test was conducted. Associations were determined using a two-tailed Spearman's rank correlation test. Figures were plotted using R version 4.0.1.

## 3 Results

### 3.1 IV BCG induces superior *M.tb*-specific antibody titers in serum compared with ID or AE BCG vaccination

**Fig. 1:**
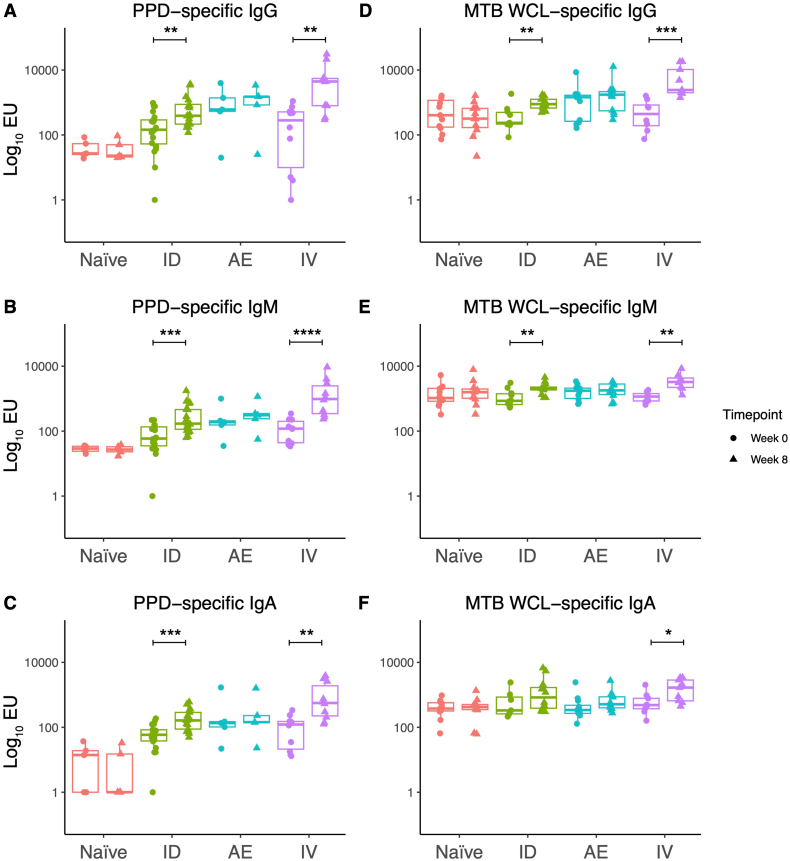
PPD-specific IgG (A), IgM (B) and IgA (C) levels in the sera of rhesus macaques collected at baseline and 8 weeks post-BCG vaccination by different routes. *M.tb* WCL-specific IgG (D), IgM (E) and IgA (F) levels in the sera of rhesus macaques collected at baseline and 8 weeks post-BCG vaccination by different routes. * denotes *p* < 0.05, ** denotes *p* < 0.01, *** denotes *p* < 0.001, **** denotes *p* < 0.0001. Bars represent the median and interquartile range (IQR).

Serum IgG, IgM and IgA responses to PPD were evaluated at baseline and 8 weeks post-vaccination in serum collected from NHPs that were unvaccinated controls or vaccinated with BCG by either the ID, IV or AE routes in Studies 1–4. Animals that received ID BCG and IV BCG vaccination showed a significant increase in PPD-specific antibody titers post-vaccination for all isotypes (Fig. 1A–C). Responses did not increase following AE BCG vaccination and the naïve unvaccinated controls had minimal levels of PPD-specific IgG, IgM, and IgA. A similar result was observed for *M.tb* WCL-specific responses, although the increase in *M.tb* WCL-specific IgA in the ID BCG vaccinated group was non-significant (Fig. 1D–F).

**Fig. 2:**
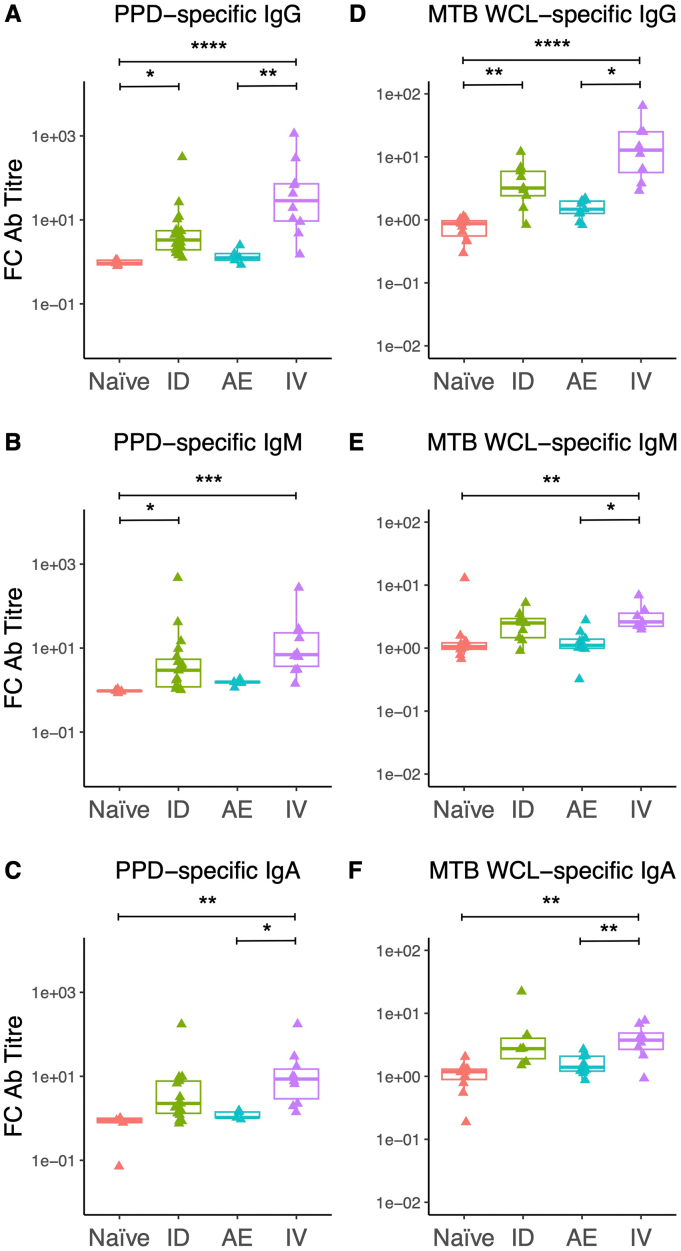
Comparison of the fold change in PPD-specific IgG (A), IgM (B) and IgA (C) levels in the sera of rhesus macaques at 8 weeks post-BCG vaccination by different routes compared with baseline. FC in *M.tb* WCL-specific IgG (D), IgM (E) and IgA (F) levels in the sera of rhesus macaques at 8 weeks post-BCG vaccination by different routes compared with baseline. * denotes *p* < 0.05, ** denotes p < 0.01, *** denotes p < 0.001, **** denotes p < 0.0001. Bars represent the median and IQR. FC = fold change, Ab = antibody.

When comparing the fold change between baseline and week 8 post-vaccination antibody responses between routes, IV BCG vaccinated animals demonstrated a superior response across all isotypes for both PPD (Fig. 2A–C) and *M.tb* WCL (Fig. 2D–F), which was statistically significant compared to that in the naïve and AE BCG groups but not the ID BCG group. There was a significant correlation between PPD-specific and *M.tb* WCL-specific IgG responses at week 8 for all groups combined (*r* = 0.93, *p* < 0.0001), for IV BCG alone (*r* = 0.73, *p* = 0.03), for ID + IV BCG (*r* = 0.82, *p* = 0.001), but not for ID BCG alone or naïve unvaccinated animals. Associations were less clear for IgM and IgA (Supplementary Fig. 1).

**Fig. 3:**
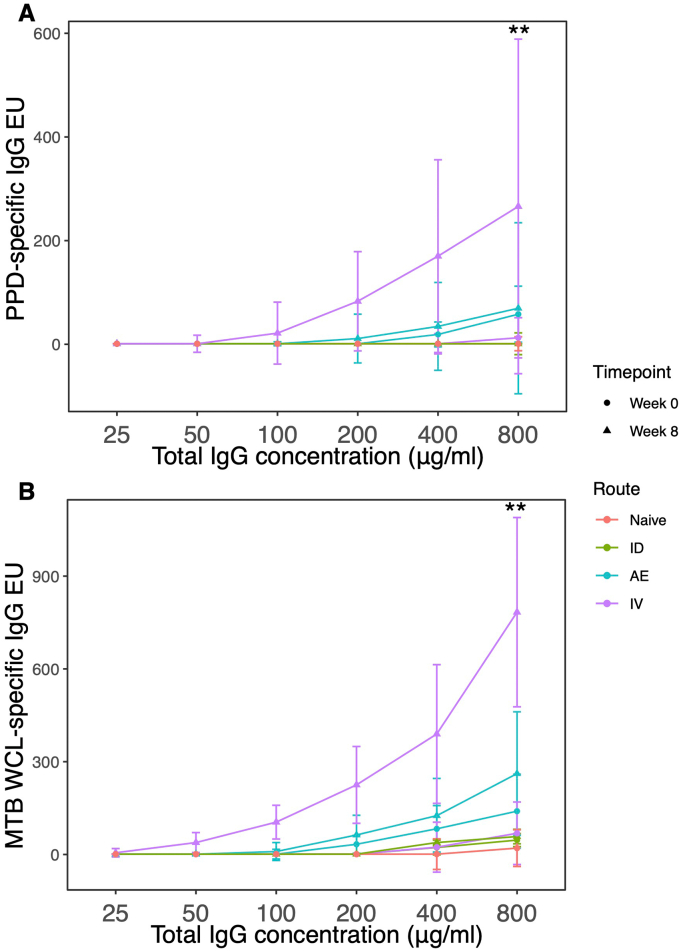
PPD-specific (A) and *M.tb* WCL-specific (B) IgG purified from serum collected at baseline and 8 weeks post-BCG vaccination by different routes. ** denotes *p* < 0.01. Bars represent the median and IQR.

Total polyclonal IgG was purified from serum collected from the same animals and levels of PPD-specific and *M.tb* WCL-specific responses were evaluated at different IgG concentrations at baseline and 8 weeks post-vaccination. Significantly higher levels of *M.tb*-specific IgG were detected following IV BCG vaccination compared with those in naïve unvaccinated animals or those that received BCG by the other routes investigated at a concentration of 800 μg/ml (Fig. 3A–B).

**Fig. 4:**
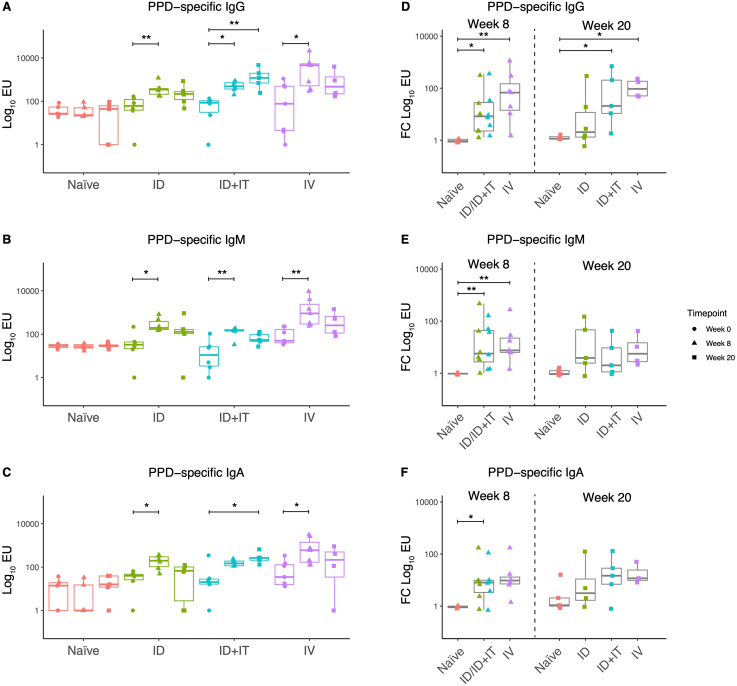
Comparison of PPD-specific IgG (A), IgM (B) and IgA (C) levels in the sera of rhesus macaques at 8 and 20 weeks post-BCG vaccination by different routes. Fold change in PPD-specific IgG (D), IgM (E) and IgA (F) levels in serum at 8 and 20 weeks following BCG vaccination by different routes relative to baseline. The ID BCG and ID + IT BCG groups are combined at 8 weeks as this is prior to the IT boost (12 weeks) and therefore both groups have received the same treatment at this timepoint. * denotes p < 0.05, ** denotes p < 0.01. Bars represent the median and IQR.

In Study 1, PPD-specific IgG, IgM and IgA responses were evaluated at baseline, week 8 and week 20 post-vaccination in serum collected from NHPs that were unvaccinated controls or vaccinated with BCG by either the ID or IV routes, or vaccinated with ID BCG with an IT boost at 11 weeks post-prime. PPD-specific IgG, IgM and IgA all showed a significant increase at week 8 post-BCG vaccination by the ID and IV routes. The animals that received an IT boost showed a significant increase in IgG and IgA (but not IgM) titer at week 20 (Fig. 4A–C). Considering fold change in antibody responses between week 8 and baseline, both ID and IV BCG vaccinated groups showed a greater IgG fold change compared with the naïve group. The fold change in IgG at week 20 was significantly greater than the naïve group for the ID+IT BCG and IV BCG (but not ID BCG) groups. Fold change in IgM at week 8 (but not 20) was significantly greater than naïve for ID and IV BCG vaccinated animals, and fold change in IgA at week 8 was significantly greater than naïve for ID BCG vaccinated animals only (Fig. 4D–F).

### 3.2 IgG induced by IV BCG vaccination has enhanced capacity to opsonize the surface of mycobacteria compared with IgG induced by ID BCG

**Fig. 5:**
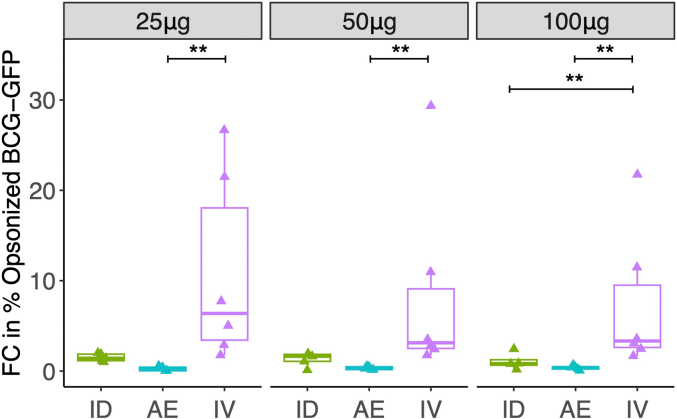
Fold change in percentage opsonization of BCG-GFP with different concentrations of IgG purified from serum collected at 8 weeks post-BCG vaccination by different routes relative to baseline. ** denotes p < 0.01. Bars represent the median and IQR.

Different concentrations of purified total IgG from animals in Studies 1–3 were evaluated for opsonizing capacity of the BCG surface at 8 weeks post-vaccination. IV BCG led to a superior fold change in IgG opsonization compared to both ID BCG and AE BCG when using 100 μg of purified IgG. However, this difference was only statistically significant between IV BCG and AE BCG (not ID BCG) when 25 μg and 50 μg of polyclonal IgG were used. A 10-fold increase in the fold change of opsonization rate of BCG-GFP was recorded for two animals vaccinated with IV BCG compared with AE BCG and ID BCG at 8 weeks post-vaccination (Fig. 5).

### 3.3 IV BCG induces higher avidity *M.tb*-specific IgG compared with ID BCG

**Fig. 6:**
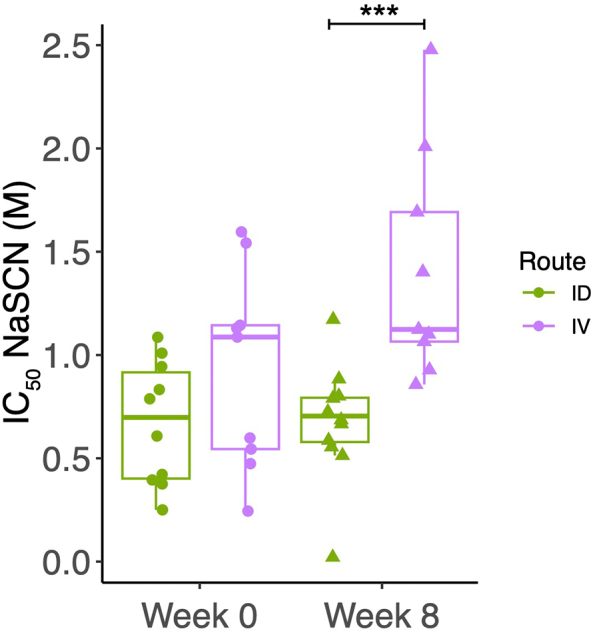
Avidity of PPD-specific IgG in serum collected at baseline and 8 weeks post-vaccination with BCG by the ID and IV routes. *** denotes *p* < 0.001. Bars represent the median and IQR.

Serum from NHPs vaccinated with BCG by the ID or IV route was evaluated at baseline and 8 weeks post-vaccination. Animals that received IV BCG vaccination showed a significant increase in PPD-specific IgG avidity following vaccination, while those that received ID BCG vaccination did not. IV BCG vaccinated animals demonstrated a superior response at 8 weeks post-vaccination compared with ID BCG vaccinated animals (Fig. 6). There was a modest but significant correlation between the titer and avidity of PPD-specific IgG at week 8 (*r* = 0.59, *p* = 0.005) (Supplementary Fig. 2).

### 3.4 *M.tb*-specific IgG avidity correlates with *ex vivo* control of mycobacterial growth and protection following *in vivo M.tb* challenge

**Fig. 7:**
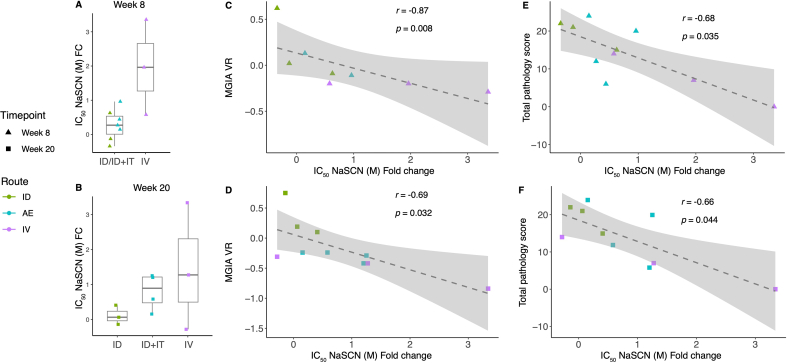
Fold change in avidity of PPD-specific IgG in serum collected at week 8 (A) and week 20 (B) post-vaccination with BCG by the ID, ID + IT and IV routes relative to baseline. The ID and ID + IT groups are combined at week 8 because at this point they had both received the same treatment (ID BCG only). The ID + IT group received an IT boost at week 12 so are plotted separately at week 20. Spearman's correlations are shown between MGIA vaccine response (VR) and fold change in avidity in PPD-specific IgG at week 8 (C) or week 20 (D). Spearman's correlations are shown between total pathology following *in vivo M.tb* challenge and fold change in avidity in PPD-specific IgG at week 8 (E) or week 20 (F). Bars represent the median and IQR.

Analysis of serum collected from animals in Study 1 that received BCG by either the ID or IV routes, or ID BCG with an IT boost at 11 weeks post-prime, revealed that neither PPD-specific IgG avidity between weeks 8 and 20 post-vaccination, not FC between groups at either time-point were significantly greater, although there was only sufficient volume of sample to perform this assay on three to four animals per group (Fig. 7A–B). As these animals were challenged with aerosol *M.tb* following vaccination, it was possible to compare avidity outcomes with pathology outcome measures post-*M.tb* aerosol challenge *in vivo* as well as *ex vivo* control of mycobacterial growth in the same animals. Fold change in IgG avidity at weeks 8 and 20 post-vaccination compared with baseline inversely correlated with the MGIA vaccine response (calculated as post-vaccination growth minus baseline growth, such that a more negative value indicates improved control) (*r* = −0.87, *p* = 0.008 and *r* = −0.69, *p* = 0.032 respectively) (Fig. 7C–D). Fold change in IgG avidity at weeks 8 and 20 post-vaccination compared to baseline also inversely correlated with total pathology score (*r* = −0.68, *p* = 0.035 and *r* = −0.66, *p* = 0.044 respectively) (Fig. 7E–F).

## 4 Discussion

Our finding that IV BCG vaccination induces superior PPD- and *M.tb* WCL-specific serum antibody titers compared with ID BCG is broadly consistent with that of Darrah et al. who showed significantly higher *M.tb* WCL-specific IgG and IgA responses in the plasma and bronchoalveolar lavage (BAL) fluid following IV BCG compared with a standard adult human dose of BCG administered ID [20]. Interestingly, unlike our study, they did not see increased levels of IgM [20]. Similarly the follow-up study by Irvine et al. noted increased PPD-specific IgG1, but not IgA or IgM, titers; although they did report significantly increased LAM-, PstS1-, Apa- and HspX-specific IgM following IV BCG vaccination compared with standard dose ID BCG [25]. It is likely that such differences can be attributed to sensitivity associated with the small sample sizes imposed on NHP studies, or to methodological differences; Darrah et al. defined an endpoint titer by ELISA [20], while Irvine et al. applied Luminex using antigen-coupled beads [25], and we used an in-house standardized ELISA. Responses to AE BCG did not increase at 8 weeks post-vaccination and were not significantly different from those induced by ID BCG, perhaps as expected when looking in the periphery. Delayed detection of cellular responses in peripheral blood has been reported following AE BCG compared with ID BCG in NHPs, so humoral immunity may similarly be delayed and a longer time-course post-vaccination could be explored [16]. Dijkman et al. also showed no increase in peripheral antibodies following mucosal delivery of BCG in NHP; rather it was IgA in the BAL fluid that increased and was identified as a correlate of local protective immunity [17]. Future studies on AE BCG-induced antibodies should focus on the lung compartment.

We chose to measure responses against *M.tb* antigen mixtures because BCG is a whole-cell vaccine and the most relevant antigenic targets for protective antibodies are currently unknown. It was interesting to note that responses to PPD and *M.tb* WCL correlated well, despite their different preparation methods which could result in denaturation of conformational epitopes in PPD from the high temperatures required. While this approach provides a broad picture of the overall antibody response, responses to different antigen fractions or specific individual antigens should be defined. Some studies report antibody titers against hypothesis-driven selected antigens following BCG vaccination [25,[27], [28], [29]], but future work should now aim to identify the most relevant targets in *M.tb* using high-throughput unbiased approaches such as whole-proteome and glycan microarrays.

We also explored the opsonizing capacity of IgG induced by different routes of BCG vaccination. Opsonization refers to the 'coating' of pathogens with antibodies which enhances their targeting and uptake by phagocytes [30]. A role for opsonizing antibodies in protection against TB has been demonstrated in mice, where opsonizing IgG monoclonal antibodies to lipoarabinomannan (LAM) on the *M.tb* cell surface conferred protection against *M.tb* infection with a dose-dependent reduction in bacterial load [31]. Furthermore, opsonization with rabbit or human antibodies specific to LAM has been shown to promote the killing of mycobacteria by human macrophages [28,32]. Chen et al. have reported opsonization of mycobacteria pre-incubated with post-vaccination sera from ID BCG vaccinated adult humans, with a significant enhancement in phagocytosis and intracellular growth inhibition when BCG was opsonized with post-vaccination compared with pre-vaccination sera [33]. However, to our knowledge the effect of IV BCG vaccination on opsonizing capacity has not been previously explored.

We demonstrate superior opsonizing capacity of IgG induced by IV BCG compared with ID or AE BCG vaccination. Future work should explore whether this translates into improved uptake and killing of mycobacteria in macrophages, as sample availability limited our ability to pursue further functional studies. We previously reported a significant correlation between opsonizing capacity using this assay and macrophage phagocytosis following ID BCG vaccination [34], suggesting that enhanced opsonization does lead to an upregulation of responses functionally relevant to mycobacterial control. Nonetheless, this approach confirms the prospective value of using flow cytometry techniques for the evaluation of opsonization of the three-dimensional surface of mycobacteria as a separate, perhaps more relevant, model to the traditional ELISA which relies on antigen preparations presented in a two-dimensional format [35,36]. It is well known that antibodies can enhance complement activation by *M.tb* and complement augments the opsonization of bacteria by antibodies [37]. It would therefore be of interest in future studies to characterize complement components in the serum from animals BCG vaccinated by different routes and their impact on opsonizing capacity.

Avidity, also known as functional affinity, measures the total binding strength of an antibody across binding sites (as opposed to affinity which measures the strength of binding at a single site) [38]. Through a process known as avidity maturation, antigen-driven selection of higher-affinity antibodies augments the magnitude and longevity of host immunity. This results from continuous antigen presentation at germinal centers to promote fine-tuning of antibody complementarity [39]. Interestingly, differences in antibody avidity have been found between patients with latent *M.tb* infection (LTBI) and active TB disease, but effects of BCG vaccination on antibody avidity have not been previously reported [40]. Our finding that BCG vaccination by the IV, but not ID, route induces an increase in *M.tb*-specific IgG avidity is novel. Affinity maturation is known to depend on the persistence of antigen in secondary lymphoid organs, which can be dependent on the adjuvant and route of immunization [41]. That IgG avidity correlates with *ex vivo* control of mycobacterial growth and protection following *M.tb* challenge in our study is of importance. Although vaccines differ in their ability to evoke avidity maturation, antibody avidity is a surrogate of protective efficacy for several vaccine types against bacterial pathogens including meningococcus, pneumococcus, and *haemophilus influenzae* [[42], [43], [44]]. Avidity may similarly represent a functionally-relevant marker for TB vaccines.

There were some limitations to our study. Sample sizes were small due to ethical limitations on the use of NHPs in research, and serum volume was often limited due to adherence with blood collection guidelines. This meant that not all assays could be performed on all samples, with our exploration of Study 1 particularly restricted. Furthermore, as the animals used in three of the four included studies were not challenged with *M.tb* following vaccination, it was not possible to relate antibody responses such as opsonizing capacity to levels of *in vivo* protection. For logistical reasons, different strains of BCG were used for the vaccinations (BCG Danish strain 1331) and the serological assays (BCG Montreal for measurement of opsonizing capacity and BCG Pasteur for MGIA). Variation between strains may influence outcomes, and ultimately it would be preferable to use *M.tb* itself to ensure clinical applicability [45].

Regarding the opsonization assay, we used mycobacteria grown in Tyloxapol to avoid clumping and resulting assay variability. It has been suggested that such detergents can alter the metabolism of mycobacteria and the expression of surface antigens in an *in vitro* setting [46,47]. However, others have shown that mAbs to α-glucan and AM/LAM bind to both BCG grown with and without detergent, and that the effect of ID BCG vaccination on opsonization of mycobacteria grown under either culture condition was unaffected [33]. It is notable that baseline antibody titers differed between groups (see Fig. 1 and Fig. 4); while we compared FC between pre- and post-vaccination time-points in an effort to overcome this, pre-existing responses may have limited ability to boost or detect further increases in some groups and not others and therefore confounded comparisons.

It would be of interest to evaluate cellular immune responses in parallel to antibody responses in the same animals, but this was beyond the scope of the study due to sample availability. Cellular responses have previously been reported for Study 1, and the frequency of PPD-specific IFN-γ secreting cells and polyfunctional CD4+ T cells was similarly higher following IV BCG compared to ID BCG vaccination, with a correlation between numbers of IFN-γ and TNF-α dual cytokine producing CD4+ T cells and reduced disease pathology [19]. Furthermore, Darrah et al. have comprehensively described cellular responses following different routes of BCG vaccination, noting that IV BCG induces substantially more antigen-responsive CD4+ and CD8+ T cells in the blood, spleen, bronchoalveolar lavage and lung lymph nodes, as well as a high frequency of antigen-responsive T cells across all lung parenchymal tissues compared with ID and/or aerosol BCG [20]. A more recent dose-ranging study has confirmed that airway T cells are a correlate of IV BCG-mediated protection against TB in NHPs [48].

### 4.1 Conclusions

In conclusion, we provide data that contributes to the recently expanding body of literature that aims to identify potential correlates of protection from TB using IV BCG vaccination in NHP as a model system. We describe for the first time the influence of IV BCG in driving enhanced *M.tb*-specific IgG avidity and opsonizing capacity, with a significant inverse association between IgG avidity and pathology following *M.tb* challenge. As discussed, Irvine et al. have previously explored antibody responses following IV BCG and describe IgM titers in the plasma and lungs of immunized macaques as markers of reduced bacterial burden [25]. It would therefore be of interest to further characterize the IgM response and determine whether avidity is similarly relevant for this isotype. Contributions of different aspects of the immune response to protection from TB are unlikely to be mutually-exclusive, and while the key to the superiority of IV BCG may lie in cell-mediated mechanisms, antibodies can act to augment such responses. Harnessing these functions through the rational design of novel TB vaccines may aid in increasing efficacy beyond that conferred by standard BCG alone.

## Funding

HMcS is a Wellcome Trust Investigator (grant code WT 206331/Z/17/Z). This research was also supported by the National Institute for Health Research (NIHR) Oxford Biomedical Research Centre (BRC). The views expressed are those of the author(s) and not necessarily those of the NHS, the NIHR or the Department of Health. RT and HMcS are Jenner Investigators. The funding sources had no involvement in study design; in the collection, analysis and interpretation of data; in the writing of the report; or in the decision to submit the article for publication.

## CRediT authorship contribution statement

**Marco Polo Peralta Alvarez:** Writing – review & editing, Writing – original draft, Visualization, Validation, Supervision, Project administration, Methodology, Investigation, Formal analysis, Data curation, Conceptualization. **Keya Downward:** Writing – review & editing, Writing – original draft, Investigation, Data curation. **Andrew White:** Writing – review & editing, Writing – original draft, Resources. **Stephanie A. Harris:** Writing – review & editing, Writing – original draft, Investigation. **Iman Satti:** Writing – review & editing, Writing – original draft, Investigation. **Shuailin Li:** Writing – review & editing, Writing – original draft, Visualization. **Alexandra Morrison:** Writing – review & editing, Writing – original draft, Investigation. **Laura Sibley:** Writing – review & editing, Writing – original draft, Investigation. **Charlotte Sarfas:** Writing – review & editing, Writing – original draft, Investigation. **Mike Dennis:** Writing – review & editing, Writing – original draft, Resources, Methodology, Investigation. **Hugo Redondo Azema:** Data curation, Formal analysis, Investigation, Methodology, Writing – original draft, Writing – review & editing. **Sally Sharpe:** Writing – review & editing, Writing – original draft, Resources, Methodology, Investigation. **Helen McShane:** Writing – review & editing, Writing – original draft, Supervision, Funding acquisition, Conceptualization. **Rachel Tanner:** Writing – review & editing, Writing – original draft, Visualization, Supervision, Project administration, Methodology, Funding acquisition, Formal analysis, Data curation, Conceptualization.

## Declaration of competing interest

The authors declare that they have no known competing financial interests or personal relationships that could have appeared to influence the work reported in this paper.

## Data Availability

Data will be made available on request.

## Appendix A Supplementary data

Supplementary material Image 1
