# Supplementary material for: Intravenous BCG vaccination in non-human primates induces superior serum antibody titers with enhanced avidity and opsonizing capacity compared to the intradermal route
Source: Vaccine. 2024 Dec 2;42(26):None. doi: 10.1016/j.vaccine.2024.126444 (PMC11906387; doi:10.1016/j.vaccine.2024.126444)
Supplement: Supplementary file 1 — Supplementary material [file mmc1.docx]

**Intravenous BCG vaccination in non-human primates induces superior serum antibody titers with enhanced avidity and opsonizing capacity compared to the intradermal route**

**Supplementary materials**


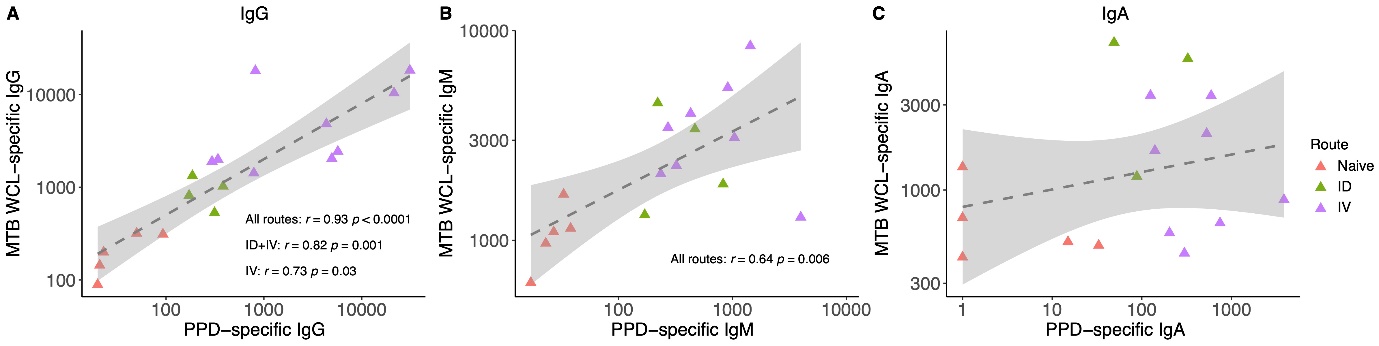


**Supplementary Figure 1.** Correlation of PPD-specific and *M.tb* WCL-specific IgG (A), IgM (B), and IgA (C) ELISA units at 8 weeks following BCG vaccination by different routes. Points represent the mean of technical triplicates.


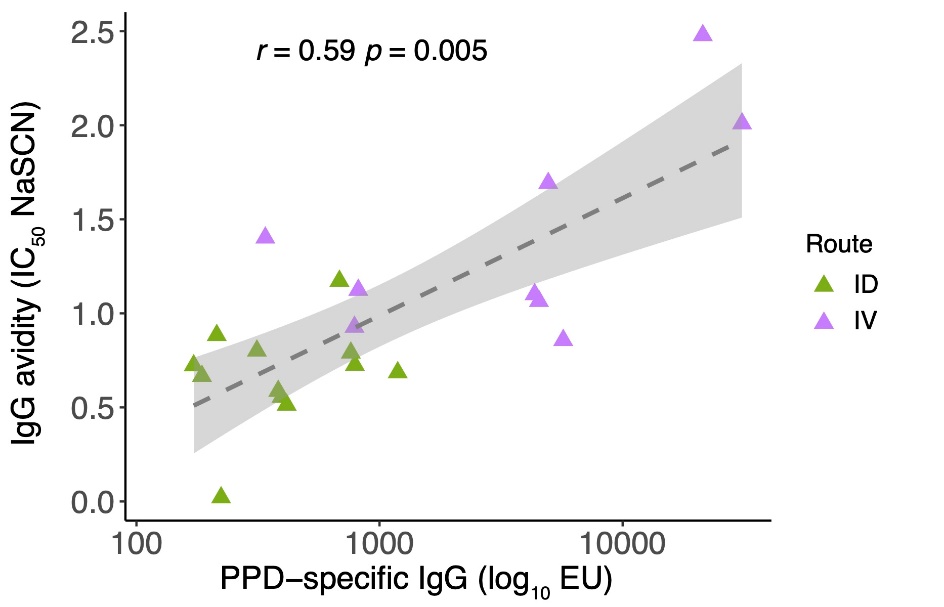


**Supplementary Figure 2.** Correlation of PPD-specific IgG ELISA units with avidity of PPD-specific IgG in serum collected at 8 weeks following BCG vaccination. Points represent the mean of technical triplicates.
